# The Pericapsular Nerve Group (PENG) block combined with Local Infiltration Analgesia (LIA) compared to placebo and LIA in hip arthroplasty surgery: a multi-center double-blinded randomized-controlled trial

**DOI:** 10.1186/s12871-022-01787-2

**Published:** 2022-08-06

**Authors:** D.-Yin Lin, Brigid Brown, Craig Morrison, Nikolai S. Fraser, Cheryl S. L. Chooi, Matthew G. Cehic, David H. McLeod, Michael D. Henningsen, Nikolina Sladojevic, Hidde M. Kroon, Ruurd L. Jaarsma

**Affiliations:** 1 Department of Anesthesiology, Flinders Medical Centre, Flinders Drive, Bedford Park, Adelaide, South Australia 5042 Australia; 2 Discipline of Perioperative Medicine, College of Medicine and Public Health, Flinders University, Adelaide, South Australia Australia; 3 Department of Orthopaedic and Trauma Surgery, Flinders Medical Centre, Adelaide, South Australia Australia; 4 Department of Surgery, Royal Adelaide Hospital, Adelaide, South Australia Australia; 5 Discipline of Surgery, Faculty of Health and Medical Sciences, School of Medicine, University of Adelaide, Adelaide, South Australia Australia; 6 Discipline of Orthopaedic Surgery, College of Medicine and Public Health, Flinders University, Adelaide, South Australia Australia

**Keywords:** Anesthesia, Analgesia, Regional analgesia, Hip arthroplasty, Sham, PENG, Pericapsular nerve group block, Pain, Patient reported outcome measures, PROMs

## Abstract

**Background:**

The PEricapsular Nerve Group (PENG) block is a novel regional analgesia technique that provides improved analgesia in patients undergoing hip surgery while preserving motor function. In this study the PENG block was investigated for analgesia in elective total hip arthroplasty (THA).

**Methods:**

In this multi-centre double-blinded randomized-controlled trial, in addition to spinal anesthesia and local infiltration analgesia (LIA), THA patients received either a PENG block or a sham block. The primary outcome was pain score (numeric rating scale 0–10) 3 h postoperatively (Day 0). Secondary outcomes were postoperative quadriceps muscle strength, postoperative Day 1 pain scores, opiate use, complications, length of hospital stay, and patient-reported outcome measures.

**Results:**

Sixty patients were randomized and equally allocated between groups. Baseline demographics were similar. Postoperative Day 0, the PENG group experienced less pain compared to the sham group (PENG: 14 (47%) patients no pain, 14 (47%) mild pain, 2 (6%) moderate/severe pain versus sham: 6 (20%) no pain, 14 (47%) mild pain, 10 (33%) moderate/severe pain; *p* = 0.03). There was no difference in quadriceps muscle strength between groups on Day 0 (PENG: 23 (77%) intact versus sham: 24 (80%) intact; *p* = 0.24) and there were no differences in other secondary outcomes.

**Conclusions:**

Patients receiving a PENG block for analgesia in elective THA experience less postoperative pain on Day 0 with preservation of quadriceps muscle strength. Despite these short-term benefits, no quality of recovery or longer lasting postoperative effects were detected.

## Introduction

Total hip arthroplasty (THA) is a cost-effective treatment for osteoarthritis through reduction in pain and improvement in quality of life [1]. It is increasingly performed in an aging population with a total of 32,929 THAs performed in Australia in 2017—2018 (133:100,000 population) [2]. THA is associated with significant postoperative pain and high rates of analgesia use, [3] with incidences of opioid prescribing following THA as high as 89.7% [4, 5].

Adequate pain management following THA is important as quality analgesia has been shown to decrease complication rates and facilitate postoperative mobilization [6, 7]. Previous THA studies have suggested a multimodal analgesia approach to decrease reliance on opioid based medications to reduce associated side-effects [3, 8]. Regional analgesia is an important part of this multimodal approach. Commonly performed regional analgesia techniques include the femoral nerve block, fascia iliaca block, or the lumbar plexus block. The major disadvantage of these regional techniques commonly used for THA is that they have only been partially effective in reducing pain and frequently result in motor weaknesses, delaying mobilization [9, 10].

In 2018, Giron-Arango et al. described a novel regional technique for hip analgesia; the pericapsular nerve group (PENG) block [11]. The PENG block is a plane block placed under ultrasound guidance at the level of the anterior inferior iliac spine, targeting the articular branches of the femoral nerve, obturator nerve, and accessory obturator nerve [12]. Randomized-controlled trials investigating the efficacy of PENG have shown improved analgesia while preserving motor function and quadriceps muscle strength, enabling postoperative mobilization and improved quality of recovery [13–15].

A common technique used for THA is spinal anesthesia in combination with local infiltrating analgesia (LIA). However, this approach is largely based on favourable results of LIA in knee arthroplasty with limited effect in postoperative pain control in THA [16]. Little is known of the addition of PENG in THA with LIA. This double-blinded randomized-controlled trial was conducted to test the efficacy of the addition of PENG in THA compared with the standard of LIA alone, using a sham block as control.

The primary outcome was the NRS pain score at Day 0. Secondary outcomes were: NRS pain score (at Day 1), Day 0 and 1 quadriceps muscle strength, perioperative opiate use, postoperative complications, length of hospital stay, patient satisfaction and PROMs.

## Methods

This multi-centre double-blinded randomized-controlled trial was conducted at two teaching hospitals in Adelaide, Australia; Noarlunga Health Services (NHS) and Flinders Medical Centre (FMC). Institutional ethics approval was obtained (SALHN/HREC/292.20) and written informed consent was acquired from all participants. The trial was registered prior to commencement (NTR; NL9147; principal investigator: D-Y.L; date of registration: 25^th^ of December 2020, URL: https://www.trialregister.nl/trial/9147). This study conforms to the Consolidated Standards of Reporting Trials (CONSORT) and the CONSORT extension for trials reporting patient-related outcomes [17, 18]. The study ran from June 28 to November 8 2021.

The inclusion criteria were adult patients presenting for primary elective THA under spinal anesthesia, without contraindications for regional analgesia, who were able to provide informed consent and reliably report symptoms to the research team. Exclusion criteria were an inability to provide first party consent (e.g. due to cognitive impairment or language barrier) and contraindications for or patient refusal of spinal anesthesia and/or regional analgesia.

### Randomization, blinding and study intervention

Patients were randomized to either PENG block (intervention) or sham block (control). Randomization was performed by the principal investigator only via an online randomization computer generator (www.sealedenvelope.com) on a 1:1 basis. Members of the surgical team, members of the Acute Pain Service (APS), nursing staff and patients were all blinded to the intervention. To ensure blinding, the anaesthesiologist performing the preoperative block was different from the anaesthesiologist managing the patient intraoperatively and conducting the postoperative assessments.

#### Block techniques

Following the administration of spinal anaesthesia, the allocated block was placed using ultrasound guidance with a curvilinear probe (2.5-5 MHz).

PENG: 20 mL of ropivacaine 0.5% (100 mg) prepared by the anaesthesiologist performing the block was used. The area was aseptically prepped and draped. The curvilinear probe was placed transversely, medial to the anterior inferior iliac spine with the medial end of the probe rotated in a caudad direction to align to the superior pubic ramus. A 100 mm sonoplex needle was inserted in-plane under ultrasound guidance. 20mLs of local anaesthetic was injected as a plane block between the psoas fascia and superior pubic rami.

#### Sham

This block was simulated by the anaesthesiologist by prepping, scanning and draping as per PENG block protocol. The probe and a blunt needle, with a 20 mL syringe filled with saline attached, were held against the skin similar to the PENG block and a sufficient pause to simulate the block being performed was conducted, without actual administration of any medicine.

Following placement of either block, a small cross was drawn with a surgical marker to cover the puncture site or absence thereof.

The study was designed to represent daily practice and to achieve high external validity. Anaesthetic technique was standardized to a spinal anaesthesia with 0.5% Isobaric bupivacaine (range 10-14 mg) without use of intrathecal opioids. A single 8 mg intravenous dose of dexamethasone was administered at the time of the block. Surgical technique was performed at the discretion of the treating orthopaedic surgeon, including routine use of LIA in all patients at a dose of 100 mL of 0.1% ropivacaine with 1 mg epinephrine. Postoperative analgesia regime was standardized with round-the-clock acetaminophen and NSAIDs if no contraindication, and if needed tramadol, oxycodone, and/or fentanyl on a nurse administered basis.

The rationale for using isobaric bupivacaine is to reflect usual practice at our institution, where the longer duration is suited to the surgery [19].

### Outcomes

#### Pain

Preoperatively, individual patient pain experience was evaluated using the Pain Catastrophizing Scale [20]. Pain scores were obtained preoperatively (baseline), 3-h postoperatively in the Recovery Unit (Day 0), and on postoperative Day 1 (16 to 22 h postoperatively, standardized), marking the maximum pain score during active movement (quadriceps muscle strength test) at each time point. Pain scores were recorded using a numeric rating scale (NRS) ranging from 0 (absence of pain) to 10 (worst pain imaginable) and grouped as no (NRS 0), mild (NRS 1–4), moderate (NRS 5–7) or severe pain (NRS 8–10).

Perioperative opiate doses were recorded preoperatively, intraoperatively, on day 0 and each postoperative day for three days with quantities converted to oral morphine equivalents. Chronic opioid use and chronic preoperative pain were defined as daily opioid use or pain interfering with activities of daily living for a duration of greater than three months.

Mobilization: Postoperatively at Day 0 once the spinal had recessed, and Day 1, a blinded anaesthesiologist assessed quadriceps muscle strength using the Oxford muscle strength grading with grouping of results into intact (5/5), reduced (1–4/5) and absent (0/5). If a patient reported reduced or absent quadriceps muscle strength, the test was carried out on the non-operative side to ensure it was not due to residual spinal effect. Day 0 measurements of dynamic pain and quadriceps strength were standardised to three hours from end time of surgery. A Timed Up-and-Go test was conducted preoperatively and on Day 1 postoperatively by physiotherapists. In this test, the patient starts in a seat at standard height, stands, walks ten feet, turns around, walks back, and sits back down [21].

#### Patient-reported outcome measures (PROMs)

Baseline preoperative anxiety and depression were noted using the validated Patient-reported outcomes measurement information system (PROMIS) anxiety and depression item banks [22]. These PROMs, along with the Pain Catastrophizing Scale, assess factors that have previously shown to influence pain experience and function [23]. Preoperatively and on Day 1, quality of recovery was evaluated using the Quality of Recovery (QoR-15) questionnaire [24].

The APS assessed patient satisfaction and pain management on Day 1 in a blinded fashion. Pain scores as a maximum on movement, quadriceps muscle strength, patient satisfaction and PROMs were collected using a scripted format. Complications throughout hospital admission, according to Clavien-Dindo classification grade, time to first mobilization and time to discharge were also recorded [25]. First mobilisation was accompanied by physiotherapy and assessment for suitability was twice a day.

The primary outcome was the NRS pain score at Day 0. Secondary outcomes were: NRS pain score (at Day 1), Day 0 and 1 quadriceps muscle strength, perioperative opiate use, postoperative complications, length of hospital stay, patient satisfaction and PROMs.

### Sample size calculation and statistical analyses

A *priori* power calculation was carried out using PASS 14 Power Analysis and Sample Size Software (Kaysville, Utah, USA) based on pain scores from a pilot study and a previous PENG randomized-controlled trial. This showed a mean pain score of 4 out of 10 points after THA on Day 0 without placement of PENG block. This was reduced to a score of 2 out of 10 points with placement of PENG block, with a standard deviation (SD) of 2 [13, 14]. A two-tailed independent-samples t-test for the difference between the two unpaired means with an alpha-error of 0.05 and power of 0.80 showed that 18 patients in each arm were required to detect a difference, 36 total. Given the high attrition rate in the pilot study, we accounted for a 40% dropout which brought numbers to 26. This was rounded up to 30.

Data collection and entry, and statistical analyses were conducted in a blinded fashion. The analysis was performed on an intention-to-treat basis using SPSS version 27 (IBM Corp., Armonk, NY, USA) and GraphPad Prism version 9 (GraphPad Software, La Jolla, Calif, USA). Parametricity of continuous variables was determined using the Shapiro–Wilk test. Normally distributed continuous variables are expressed as mean (SD), and nonparametric variables as median (range). Univariate analysis was carried out using the chi^2^ test or Fisher’s exact test (for *n* < 10) for categorical variables, and the Mann–Whitney U-test for nonparametric continuous variables or the Student’s t-test for parametric continuous variables. A *p*-value of < 0.05 was considered statistically significant.

## Results

**Fig. 1:**
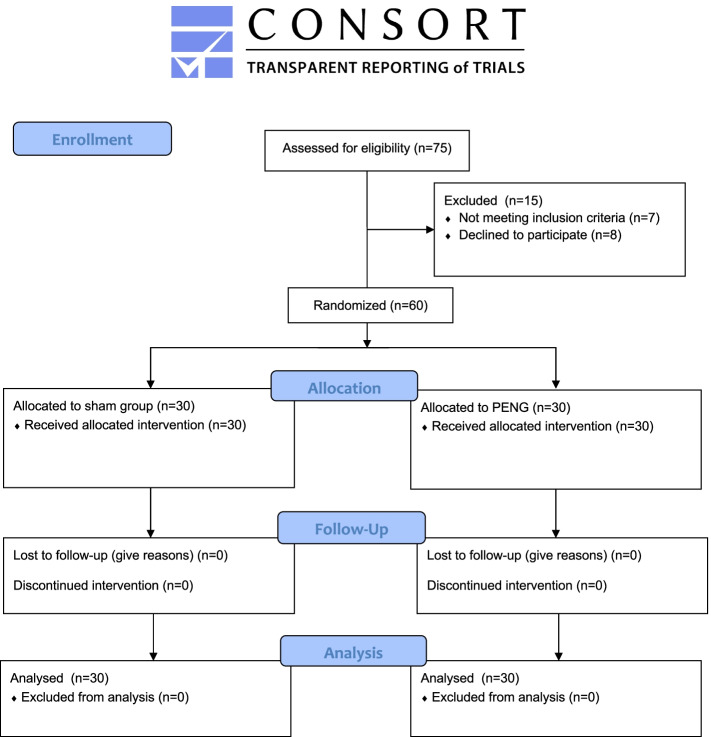
CONSORT flow diagram

During the study period, 75 patients were admitted for elective THA and screened for eligibility. Seven patients were excluded on the basis of cognitive impairment or a language barrier. Eight patients declined to participate, due to a preference for general anesthesia instead of the standardized spinal anesthesia, leaving 60 patients who were consented and randomized equally between both groups. (Fig. 1) All patients completed the study and were included in the final intention to treat analysis without loss to follow up.

The preoperative demographics of both groups were similar, including baseline NRS pain scores, pain catastrophising scores, incidence of chronic pain and anxiety or depression. (Table 1).

**Table 1:** Patient and preoperative characteristics

	**Sham** (*n* = 30)	**PENG** (*n* = 30)	***P*-value**
Age in years, mean (± SD)^a^	68.3 (± 10.9)	68.6 (± 9.5)	0.91
Gender, n (%)^b^			
Male	14 (47)	13 (43)	0.80
Female	16 (53)	17 (57)	
Weight in kg, mean (± SD)^a^	84.8 (± 20.8)	88.6 (± 21.9)	0.51
BMI in kg/m^2^, median (IQR)^c^	30.8 (27.5–32.8)	33.2 (28.3–36.7)	0.09
Mobility, n (%)^b^			
Independent (no aids)	16 (53)	10 (33)	0.13
Assisted (stick/walker/ wheelchair)	14 (47)	20 (67)	
Residence, n (%)^d^			
Home	30 (100)	29 (97)	1.00
Assisted living	0 (0)	1 (3)	
ASA score, n (%)^b^			
I	0 (0)	0 (0)	0.14
II	19 (63)	13 (43)	
III	10 (33)	17 (57)	
IV	1 (3)	0 (0)	
History of anxiety and/or depression, n (%)^d^			
Yes	7 (23)	3 (10)	0.30
No	23 (77)	27 (90)	
Chronic pain, n (%)^d^			
Yes	30 (100)	28 (93)	0.49
No	0 (0)	2 (7)	
Preoperative pain score (NRS**)**, n (%)^b^			
None (0)	0 (0)	0 (0)	0.44
Mild (1–4)	0 (0)	0 (0)	
Moderate (5–7)	7 (23)	3 (10)	
Severe (8–10)	23 (77)	27 (90)	
Preoperative pain score (NRS), median (IQR)^c^	8 (7.8–10)	9 (8–10)	0.20
Preoperative Pain Catastrophising Scale Score median (IQR)^c^	14.5 (6.75–38)	17 (6–41)	0.72
Preoperative PROMIS Depression T Score, median (IQR)^c^	52.3 (37.1–61.2)	53.3 (37.1–62)	0.88
Preoperative PROMIS Anxiety T Score, median (IQR)^c^	49.4 (37.1–58.4)	47.7 (37.1–60.7)	0.74
Chronic opiate use, n (%)^d^			
Yes	7 (23)	9 (30)	0.56
No	23 (77)	21 (70)	
Gabapentinoid use preoperatively, n (%)^d^			
Yes	3 (10)	3 (10)	1.00
No	27 (90)	27 (90)	
Operative side, n (%)^b^			
Left	14 (47)	14 (47)	1.00
Right	16 (53)	16 (53)	
Surgical procedure, n (%)^d^			
Non-cemented total	9 (30)	8 (27)	0.77
Cemented total	21 (70)	22 (73)	
Surgical approach, n (%)^b^			
Direct anterior	15 (50)	18 (60)	0.44
Posterior	15 (50)	12 (40)	
Type of anaesthesia for surgery, n (%)^d^			
General	1 (3)	1 (3)	1.00
Spinal	29 (97)	29 (97)	

*Abbreviations*: *IQR* Interquartile range, *SD* Standard deviation, *PENG* Pericapsular nerve group block, *NRS* Numeric rating scale, *PROMIS* Patient-Reported Outcomes Measurement Information System

^a^ Student’s t-test used

^b^ Chi^2^ test used

^c^ Mann–Whitney U-test used

^d^ Fisher’s exact test used

### Primary outcome

Day 0 pain scores in PENG block patients were significantly lower than in the sham block group: 14 patients (47%) in the PENG group reported no pain, compared to 6 patients (20%) in the sham group (*p* = 0.03). In both groups, 14 patients (47%) reported mild pain, and 2 patients (6%) in the PENG group experienced moderate or severe pain, compared to 10 patients (33%) in the sham group. (Table 2) These pain scores were maximum and on mobilisation, as quadriceps strength was tested immediately prior.

**Table 2:** Postoperative pain and motor outcomes

	**Sham** (*n* = 30)	**PENG** (*n* = 30)	***P*-value**
Maximum postoperative pain score (NRS) in Recovery Unit (Day 0), n (%)^a^			
None (0)	6 (20)	14 (47)	**0.03**
Mild (1–4)	14 (47)	14 (47)	
Moderate (5–7)	9 (30)	2 (6)	
Severe (8–10)	1 (3)	0 (0)	
Quadriceps muscle strength in Recovery Unit (Day 0), n (%)^a^			
Intact	24 (80)	23 (77)	0.24
Reduced	4 (13)	7 (23)	
Absent	2 (7)	0 (0)	
Maximum postoperative pain score (NRS) on Day 1, n (%)^a^			
None (0)	2 (6)	1 (3)	0.82
Mild (1–4)	8 (27)	7 (23)	
Moderate (5–7)	12 (40)	11 (37)	
Severe (8–10)	8 (27)	11 (37)	
Quadriceps muscle strength on Day 1, n (%)^a^			
Intact	22 (73)	24 (80)	0.75
Reduced	6 (20)	5 (17)	
Absent	0 (0)	0 (0)	
Unable to assess	1 (3)	1 (3)	

*Abbreviations*: *PENG* Pericapsular nerve group block, *NRS* Numeric rating scale

^a^ Chi^2^ test used

### Secondary outcomes

On Day 1, pain scores were similar between both groups (*p* = 0.82). Quadriceps muscle strength was preserved in the PENG group and was similar when compared to the sham block group on Day 0 (*p* = 0.24) and Day 1 (*p* = 0.75): On Day 0, 23 (77%) PENG patients and 24 (80%) sham block patients had intact quadriceps muscle strength (*p* = 0.24), and on Day 1 this was 24 (80%) and 22 (73%) respectively (*p* = 0.75). (Table 2).

Complication rates were similar between both groups. One patient in the sham group had uncontrolled postoperative pain on the ward, requiring maximalisation of oral analgesia, commencement of a fentanyl patient-controlled analgesia pump, and at the end of Day 1 placement of a PENG block. (Table 3). These measures were largely effective. This patient was regarded as a sham patient as per intention-to-treat, and the primary and most secondary outcome measures had already been collected.

**Table 3:** Other (post)operative outcomes

	**Sham** (*n* = 30)	**PENG** (*n* = 30)	***P*-value**
Length of operation in minutes, mean (± SD)^a^	108.07 (± 21.3)	105.57 (± 28.7)	0.70
Time to first mobilization in minutes, median (range)^b^	1450 (1263.5–1592.5)	1374 (1257.5–1560)	0.30
Time to discharge in days, median (range) ^b^	2 (1.75–3)	2 (1–3)	0.97
Clavien-Dindo complication grade, n (%)^c^			
0	24 (80)	25 (83)	0.55
I	4 (13)	5 (17)	
II	0 (0)	0 (0)	
III	1 (3)	0 (0)	
IV	1 (3)	0 (0)	
V	0 (0)	0 (0)	
Complications, n (%)			
Wound infection	0 (0)	0 (0)	N/A
Reoperation	0 (0)	0 (0)	
STEMI/NSTEMI	1 (3)	0 (0)	
Extreme postoperative pain	1 (3)	0 (0)	
Death	0 (0)	0 (0)	

*Abbreviations*: *N/A* Not applicable, *PENG* Pericapsular nerve group block, *STEMI* S-T elevation myocardial infarction, *NSTEMI* Non S-T elevation myocardial infarction

^a^ Student’s t-test used

^b^ Mann–Whitney U-test used

^c^ Chi^2^ test used

There were no differences in PROMs, Timed Up-and-Go tests, patient satisfaction, time to first mobilization, time to discharge and postoperative opiate use between groups. (Tables 4 and 5).

**Table 4:** Patient outcome questionnaires and Timed Up-and-Go tests

	**Sham** (*n* = 30)	**PENG** (*n* = 30)	***P*-value**
Preoperative QoR-15, mean (± SD)^a^	107 (± 20.6)	99.1 (± 27.4)	0.22
Postoperative QoR-15, mean (± SD)^a^	103 (± 22.8)	96.6 (± 13.6)	0.19
Timed up-and-go in seconds, preoperative, n (%)^b^			
0–15	12 (40)	9 (30)	0.61
16–30	7 (23)	8 (27)	
31–45	4 (13)	5 (17)	
46 +	2 (7)	5 (17)	
Unable to perform	5 (17)	3 (9)	
Timed up-and-go in seconds, postoperative on Day 1, n (%)^b^			
0–15	0 (0)	1 (3)	0.58
16–30	11 (37)	9 (30)	
31–45	9 (30)	10 (33)	
46 +	9 (30)	8 (27)	
Unable to perform	1 (3)	2 (7)	
Patient satisfaction, n (%)^b^			
Unsatisfied	1 (3)	1 (3)	1.00
Satisfied	23 (77)	23 (77)	
Ambivalent	6 (20)	6 (20)	

*Abbreviations*: *SD* Standard deviation, *IQR* Interquartile range, *PENG* Pericapsular nerve group block, *QoR-15* Quality of Recovery 15, *SD* Standard deviation

^a^ Student’s t-test used

^b^ Chi^2^ test used

**Table 5:** Postoperative opiate use

	**Sham** (*n* = 30)	**PENG** (*n* = 30)	***P*-value**
Postoperative opiate use in morphine equivalents (mg), median (IQR)^a^			
Day 0	30 (18.9–73.0)	30 (8.0–57.5)	0.31
Day 1	49 (21.0–93.3)	46 (15.0–73.2)	0.41
Day 2	30 (0–47)	8 (0–45.0)	0.24
Day 3	0 (0–15)	0 (0–8.0)	0.81
Total	122 (56.5–232.5)	97.5 (30.5–164.3)	0.23

*Abbreviations*: *PENG* Pericapsular nerve group block, *mg* Milligrams, *IQR* Interquartile range

^a^ Mann–Whitney U-test used

### Adverse events and protocol deviations

In two patients, one in each group, it was technically not possible to perform a spinal anaesthesia. Both had multiple failed attempts at locating a vertebral interspace for neuraxial injection. Therefore, both received a general anaesthetic for surgery.

## Discussion

This double-blinded randomized-controlled trial shows that the PENG block significantly reduces short-term postoperative pain in elective THA when spinal anaesthesia and LIA are used. (*p* = 0.03). The direct postoperative analgesic advantage of the PENG block in this setting does not remain after surgery on Day 1.

Regional analgesia in THA has traditionally been performed using a femoral nerve or fascia iliaca block. Although partially effective, these blocks result in a decrease in muscle strength [26]. Since the PENG block affects only the articular branches of the femoral and accessory obturator nerves, it is believed to achieve adequate analgesia while also preserving motor function and muscle strength. In the current study, postoperative quadriceps muscle strength was similar in both groups. This allows patients to mobilize early following surgery, which, in itself is associated with fewer complications, shorter length of hospital stay and lower mortality [27–29]. Patients who received the PENG block were thus able to mobilize as soon as the sham group patients, with less pain.

The motor sparing effect is consistent with previous studies focused on anatomy suggesting that the PENG block targets the articular branches of the femoral, obturator, and accessory obturator nerves [12]. It must be mentioned that on Day 0 and Day 1, respectively seven and six PENG patients did experience reduction in quadriceps muscle strength, however, this incidence was similar in the sham group (6 and 7 patients respectively; *p* = 0.24 and *p* = 0.75). This could reflect a reluctance to actively move the newly operated hip, or possible spread from the LIA to the femoral nerve, consistent in both groups. Notably, no adverse events directly related to block placement were reported and patient satisfaction was similar across both groups.

A variety of PROMs and outcome measures were used with the aim to objectively quantify possible recovery benefits of the PENG block. Preoperative patient PROMs, quantified using the Pain Catastrophizing Scale, PROMIS anxiety and depression item banks, were all similar between groups. Postoperative PROMs, quality of recovery and the Timed Up-and-Go tests were also similar. This could possibly be due to the timing of these tests on Day 1 postoperatively, after the analgesic effect of the PENG block had finished. A recent RCT comparing PENG to sham in combination with intra-articular injection also showed only short term benefit, without differences in longer term outcomes [30].

The similar opiate use in both groups, despite a difference in pain scores, may be due to the advanced age of the included patients and their low baseline opiate use. It is also important to note that the study was not powered to detect a difference in opiate use nor in PROMs between both groups, for which larger studies will be required to investigate this in the future.

### Limitations

Some limitations have to be addressed. As indicated above, this trial was conducted on a relatively small number of patients and could not identify differences in secondary outcomes. However, it was powered on the primary outcome, showing a significant difference between both groups.

Quadriceps strength was measured by a blinded clinician. A standardised dynamometric measurement tool would have been more accurate, but this was not available. We recognise that this makes the secondary outcome less reliable due to interobserver variation, but have addressed this by grouping the intermediate scores together.

Due to the standardized spinal anaesthesia in the study protocol, 11% (8/75) patients approached, chose not to participate, potentially inflicting some selection bias. However, randomization took place after inclusion to reduce this bias. In the future, a next randomized-controlled trial to further investigate the efficacy of PENG block in THA patients could therefore be in patients having either spinal or general anesthesia.

## Conclusion

Patients receiving an additional PENG block for analgesia during total hip arthroplasty experience less direct (Day 0) postoperative pain, with preserved quadriceps muscle strength and similar time to mobilization compared to patients having spinal anesthesia and local infiltration analgesia only. For total hip arthroplasty, the PENG block should be considered as part of multimodal analgesia.

## Data Availability

Available upon reasonable request. D-yin.lin@sa.gov.au.

## Abbreviations

THA: Total hip arthroplasty
PENG block: Pericapsular nerve group block
RCT: Randomized controlled trial
NRS: Numeric rating scale
NHS: Noarlunga Health Services
FMC: Flinders Medical Centre
NTR: Netherlands Trial Registry
CONSORT: Consolidated Standards of Reporting Trials
APS: Acute Pain Service
LIA: Local infiltration analgesia
PROMs: Patient reported outcome measures
PROMIS: Patient-reported outcomes measurement information system
QoR-15 questionnaire: Quality of Recovery-15 questionnaire
SD: Standard Deviation
